# Indirect Effect of African Swine Fever on the Diet Composition of the Gray Wolf *Canis lupus*—A Case Study in Belarus

**DOI:** 10.3390/ani11061758

**Published:** 2021-06-12

**Authors:** Daniel Klich, Grigorij Yanuta, Maria Sobczuk, Marek Balcerak

**Affiliations:** 1 Department of Animal Genetics and Conservation, Warsaw University of Life Sciences-SGGW, Ciszewskiego 8, 02-786 Warsaw, Poland; daniel_klich@sggw.edu.pl (D.K.); maria_sobczuk@sggw.edu.pl (M.S.); 2 Department of Animal Breeding, Warsaw University of Life Sciences-SGGW, Ciszewskiego 8, 02-786 Warsaw, Poland; marek_balcerak@sggw.edu.pl

**Keywords:** ASF, epidemic, gray wolf, wild boar, deer, elk, beaver, diet, Belarus

## Abstract

**Simple Summary:**

The wild boar population decreased drastically in Eastern Europe after the emergence of a viral disease called African Swine Fever. We studied how the gray wolves’ diet changed in two regions in Belarus during this situation. Wolves mainly hunted wild boar, elk, red deer, roe deer and beaver. The decrease in the wild boar population caused changes in the diet of wolves, but only in Vitebsk region. After the decrease in the wild boar population, wolves in this region hunted wild boar less, but they hunted roe deer and red deer more. The more the wolves consumed wild boar, the less they consumed both deer species (roe deer and red deer). Moreover, the more the wolves consumed elk, the less they consumed beaver. In another region, Grodno, no changes in the wolves’ diet were found.

**Abstract:**

After the emergence of African swine fever (ASF), the wild boar population numbers fell drastically in Eastern Europe. This situation made it possible to verify the changes in the wolves’ diet that occurred. The material collection was carried out in two regions, Grodno and Vitebsk, in Belarus. In total, 19 species/groups of prey were observed in the gray wolf diet, but the most important were wild boar, elk, red deer, roe deer and beaver. The decrease in the number of wild boar caused changes in the diet of wolves but only in Vitebsk region, where wolves’ diet before the ASF epidemic outbreak consisted mainly of elk and wild boar. After the decrease of wild boar numbers, wolves still mainly hunted elk, but other types of prey included roe deer, red deer and beaver. We found a negative correlation between wild boar and both deer species (roe deer and red deer) in the wolves’ diet. Moreover, the more the wolves consumed elk, the less they consumed beaver. In our opinion, only intensive hunting of wolves by humans can explain the resulting dietary fluctuations between elk and beaver, as well as the fact that wolves did not turn to other food sources.

## 1. Introduction

Gray wolf (*Canis lupus*) is the most common large carnivore in Europe. The population dynamics of this species can significantly affect the dynamics and distribution of certain groups of ungulates in ecosystems [1,2]. This species is a carnivorous opportunist with a wide choice of prey [3,4,5,6,7]. We can talk, however, about a dietary preference when a predator hunts a given species of prey disproportionately to its abundance in the environment [8]. Former studies on the gray wolf’s diet show a clear preference for some species of prey [9,10]. Moreover, preferences for selected age classes of prey have also been found: young ungulates under the age of one year often fall prey to wolves [11,12,13]. The gray wolf’s main prey species are red deer (*Cervus elaphus*), elk (*Alces alces*), wild boar (*Sus scrofa*) and livestock [7,9,14]; however, depending on the availability of prey, gray wolves sometimes feed on fish and rodents, but large and medium-sized animals usually predominate in their diet.

As a consequence of prey density differences, a wolf’s diet can vary significantly between regions and habitat types. Wolves mainly hunt elk and deer in Scandinavia and northern North America [15,16,17,18]; red deer, wild boar, roe deer and beaver in Central Europe [19,20,21]; livestock in Greece [22]; and livestock, wild boar and roe deer in Italy [23,24]. When a given species dominates in the gray wolves’ diet, a decrease in its density significantly changes the composition of the wolves’ diet. Such a relation was found for wild boar, whose fall in numbers caused it to be replaced by medium-sized ungulates of the deer family [25]. This was also confirmed for beaver, for which a decline in the number of ungulates caused an increase in predation on this species [26,27]. Diet fluctuations have been observed even without significant fluctuations in the number of individual ungulate species [28]. Within one region, the proportion of wild boar in the diet can vary significantly [29].

Depending on local conditions, wild boar might be second- or third-order prey, and the share of this species in the biomass consumed by wolves can exceed 20% [19,30]. Wild boar may also be the most important prey when a decrease in the numbers of other ungulate species is observed [23]. However, after the emergence of African swine fever (ASF), which is a viral swine disease [31], the wild boar population numbers changed drastically in Eastern Europe. The virus appeared in 2013 in Belarus and caused (mainly as a result of deliberate intensive hunting) a drastic decline of the wild boar population, which is one of the gray wolf’s main prey [32,33]. The number of wild boar fell by 90% over a short period of time [34]. This situation, which is unique to Europe, made it possible to verify changes in the wolves’ diet as a result of a drastic decline in food availability. A recent study in Estonia showed a shift in the wolves’ winter diet to a higher proportion of roe deer and other less typical food sources after the emergence of ASF [35]. We chose two regions in Belarus whose densities of ungulates and species compositions differed [36]. We hypothesized that due to the drastic wild boar population decline, the wolves’ diet would shift to other ungulate species and other less-hunted animals.

## 2. Materials and Methods

### 2.1. Study Areas

**Figure 1:**
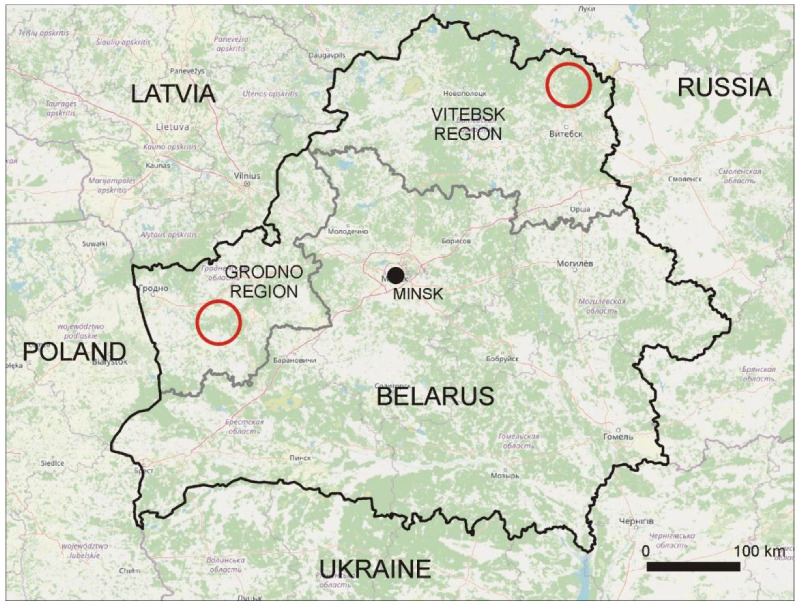
Location of study sites (red circles) in the studied regions.

The material collection was carried out in two regions, Grodno and Vitebsk, both of which are in Belarus (Figure 1). These regions were chosen because the abundance of wolves’ prey (mainly roe deer and red deer) differs between them [36]. Roe deer density was over four times higher in the Grodno region than in Vitebsk (18.7 and 4.3 ind./10 km^2^, respectively). Red deer was not noticed in the Vitebsk region, while in the Grodno region, the density was estimated to be 5.1 ind./10 km^2^. The Vitebsk region is located in north-eastern Belarus and covers two districts: Gorodok and Vitebsk (55°23′ N 30°16′ E). The study area was characterized by vast forests and swamps with a dense network of natural water bodies: rivers, streams and glacial lakes. Forest complexes covered about 72% of the area. Broad-leaved tree species prevailed (52%), and coniferous tree species covered about 44% of the area. The remaining 28% of the region was open areas, mainly agricultural lands (meadows and crop fields). The Grodno region is located in western Belarus and contains Mostovsky and Grodno districts (53°25′ N 24°48′ E). The region was mainly open areas (63%). Forest complexes that were dominated by pine (*Pinus sylvestris*) (59%) and birch (13%) stands covered about 37% of the region.

### 2.2. Population Trends

Based on official data [34], wild boar population numbers in Belarus underwent a drastic decline between 2013 and 2014 (Table 1). From 2010 to 2013, the population grew and reached 80,000 individuals. In 2014, only 8600 individuals were registered in official data. Subsequently, the population numbers of wild boar constantly decreased to 2400 individuals in 2019. Data for 2020 were not yet available. Wild boar hunting almost doubled between 2010 and 2013, and from 2014, the harvest numbers exceeded the estimated population size.

The beaver population trends show that the population density was rather stable (Table 2). In the Vitebsk region, a slight decrease was observed between 2010 and 2019 (from 13.3 to 10.1 ind./10 km^2^). In the Grodno region, the beaver density was much lower than in Vitebsk region, but only slight fluctuations were observed during the studied period.

The official data did not cover ungulates in the study sites or regions, and the population trends were not clearly known. Nevertheless, changes in the ungulate population density could be derived from the census conducted by Yanuta (unpublished data), for which typical winter track counts on transects (34–37 km for each year) were conducted, according to Priklonski [38]. The ungulate population trends could be regarded as stable, except for wild boar (Table 3). In both regions, wild boar density declined after the ASF outbreak (years: 2015–2018). Other ungulate populations were stable, and a slight increase of red deer in Grodno was observed.

### 2.3. Sample Collection, Elaboration and Statistics

We collected 237 gray wolf fecal samples from both regions between 2010 and 2020: 116 in the Vitebsk region and 121 in the Grodno region (Table 4). We tried to collect a similar number of samples for particular periods of the year. The samples were collected during both vegetation and non-vegetation periods in both regions: April to October (summer) and November to March (winter). The collection of excrement was carried out where wolves had marked their territory, which was revealed in the winter. No more than two feces samples were collected from each site marked by wolves during a given season, to assure collection of samples from various wolf packs. The samples were placed in plastic bags with detailed labels. Subsequently, the samples were immediately analyzed or frozen.

The identification of prey species in the fecal samples was performed according to Jędrzejewska and Jędrzejewski [39], Pucek [40] and Debrot et al. [41]. A washed sample was dried, and the mass of the residues was weighed. The relative amount of prey was calculated from the dry mass of the undigested residues in the sample. To estimate the biomass of individual prey, the weight of the residues of prey extracted from the feces was multiplied by digestibility coefficients [42]. Transverse hair microstructure analysis was used to identify taxonomic groups [43].

As the percentage of each prey species varied significantly between samples, we calculated the mean percentage of each prey species from all samples from each year in a given region. In further analyses, we included the main prey species: wild boar, red deer, roe deer, elk, beaver and others (as a sum of the percentage of all other prey). To show the changes in the diet composition, we calculated the mean percentage of each prey in the wolves’ diet for the three years before the ASF epidemic outbreak (2010–2013) and for three (for the Vitebsk region, 2017–2020) or two (for the Grodno region, 2017–2019) years after the epidemic outbreak. We compared the wolves’ diet between periods using the Z-test, with which the frequency of each species in the samples was analyzed. Each pray species was compared separately for the given region. We also built 12 linear regression models (6 for each region); for each model, the dependent variable was the percentage of each prey, and the explanatory variable was the year. The models were built to show the fluctuations in the percentage of each prey in the wolves’ diet for each year. We also ran a Pearson’s correlation matrix, which tested the relations among the percentage of prey species in the wolves’ diet. All statistics were calculated in SPSS software.

## 3. Results

In the wolf diet in the two regions, we observed 19 species/groups of prey in total. Apart from wild boar, elk, red deer and beaver, other less important species/groups were observed: red fox (*Vulpes vulpes*), muskrat (*Ondatra zibethicus*), white hare (*Lepus timidus*), brown hare (*Lepus europaeus*), rodents, birds, domestic dogs and livestock (cows and sheep).

**Figure 2:**
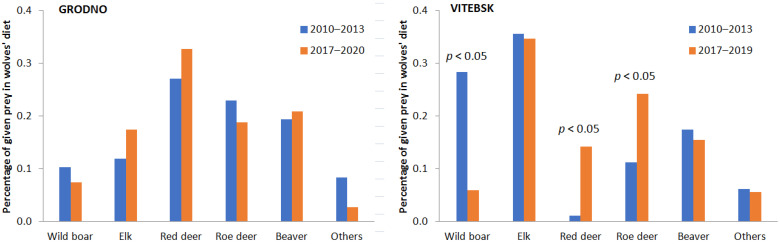
Percentage of prey in wolf diet before (2010–2013) and after (2017–2020 for Grodno and 2017–2019 for Vitebsk) the ASF epidemic outbreak in the two studied regions; statistical difference (Z-test) between periods is shown above the bars.

In Grodno, before the ASF epidemic outbreak, the main prey species were red deer, roe deer and beaver (27.0%, 22.9% and 19.4%, respectively). Wild boar and elk were less hunted by wolves (10.3% and 11.9%, respectively). This distribution did not change much after the ASF epidemic outbreak. Red deer, roe deer and beaver were still the main prey species (32.7%, 18.8% and 20.9%, respectively), but elk was more hunted (17.4%), while the share of wild boar in the wolves’ diet decreased slightly (7.4%) (Figure 2). The proportion of each species in the diet did not differ statistically between the periods (*p* > 0.05).

In the Vitebsk region, the wolves’ diet before the ASF epidemic outbreak was dependent mainly on elk and wild boar (35.6% and 28.4%, respectively). Other species were less hunted, mainly beaver (17.4%) and roe deer (11.6%), and red deer was killed only extremely rarely (1.1%). After the ASF epidemic outbreak, the diet composition changed significantly. Wolves still mainly hunted elk (34.7%), but other prey species were roe deer, beaver and red deer (24.2%, 15.4% and 14.2%, respectively). The proportion of wild boar in the diet dropped to 5.9% (Figure 2). The Z-test showed a statistically significant lower proportion of wild boar after the ASF epidemic outbreak (*p* < 0.05). Roe deer and red deer proportions were statistically higher in the period after the ASF epidemic outbreak (*p* < 0.05).

**Figure 3:**
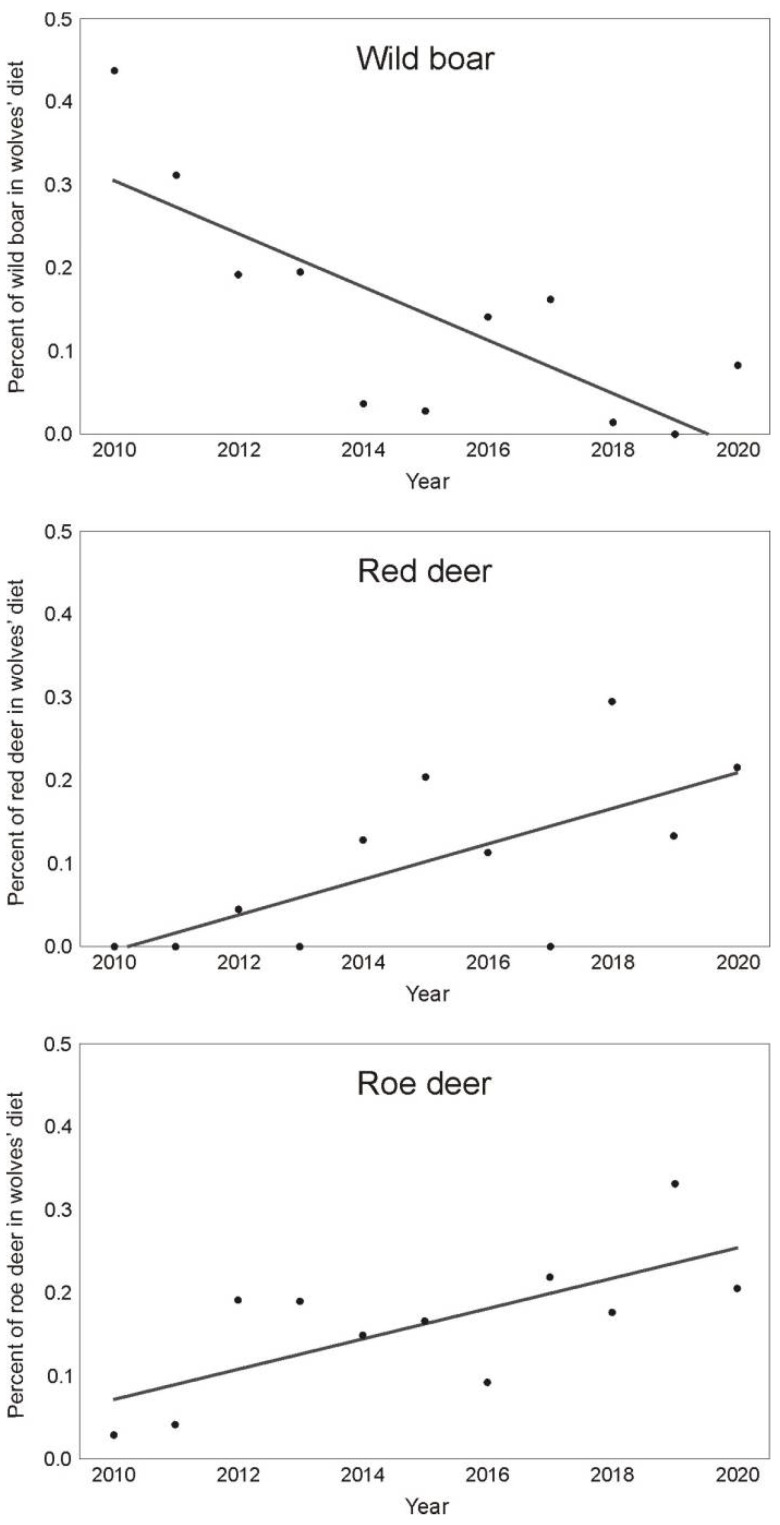
The fluctuating proportions of wild boar, red deer and roe deer in the wolves’ diet over time in the Vitebsk region.

In the Grodno region, no change was found in any prey species in the wolves’ diet over time. All the regression coefficients were statistically non-significant (*p* > 0.05). In the Vitebsk region, we found three statistically significant relations with year (Figure 3). The share of wild boar in the wolves’ diet decreased over time (F = 13.78, *p* = 0.005), which explained over 60% percent of the variance of wild boar in the gray wolves’ diet (R^2^ = 0.605). A percentage increase of two other species in the wolves’ diet was found over time for which the explanatory power was higher: red deer (F = 7.82, *p* = 0.021, R^2^ = 0.465) and roe deer (F = 8.96, *p* = 0.015, R^2^ = 0.499). No other changes over time were found.

**Figure 4:**
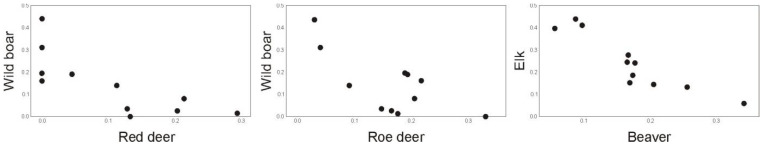
Correlation of the share in the wolves’ diet of selected prey species in the Vitebsk region.

We found no correlation between prey species in the wolves’ diet in the Grodno region. In the Vitebsk region, we found a strong correlation between the percentage in the wolves’ diet of wild boar and red deer (r = −0.767, *p* = 0.006), and between wild boar and roe deer (r = −0.714, *p* = 0.014). Elk did not correlate with wild boar (*p* > 0.05), but it strongly correlated with beaver (r = −0.907, *p* = 0.000). All relations were negative (Figure 4).

## 4. Discussion

According to our hypothesis, along with the decline of the wild boar population, the wolves increasingly hunted other ungulate species, such as roe and red deer. This was observed in the Vitebsk region, where these species were much less important in the wolves’ diet before the ASF epidemic outbreak. The strong correlation between wild boar and roe and red deer confirmed this finding (Figure 4). This result was in line with other studies that reported a change in the proportion of ungulates (roe deer, red deer and wild boar) in the diet of the gray wolf. In relatively natural ecosystems with an abundance of ungulates of the deer family, the share of wild boar in the wolf’s diet is usually low [9,14]. Ansorge et al. [44] observed that when roe deer and red deer numbers increased, wolves usually hunted them more than wild boar. However, when the numbers of ungulate prey species fell, wolves instead hunted medium-sized ungulates, mainly wild boar [23,25]. Thus, in some regions, the wolves’ diet may be based on wild boar [45,46,47], and this species’ offspring may even seasonally dominate in the diet, despite the high density of other ungulate species [48]. In contrast to the results in the Vitebsk region, the wolves did not show any response to the decline in the wild boar population in the Grodno region. This was an effect of the low share of this species in the wolves’ diet before the ASF epidemic outbreak (only 10.3%). The observed changes in the wolves’ diet were usually related to the decline of prey species that are an essential group exploited by wolves.

Contrary to our hypothesis, the decline in the wild boar population did not cause an increase of other prey in the wolves’ diet, except for ungulates. Wolves did not hunt domestic animals more often, nor did they turn to any other food source. This result was surprising, as some authors have indicated that the most important buffer victims that compensate for a deficit of ungulates are medium-sized wild mammals (mainly hares, but also raccoons, dogs and beavers) and domestic animals (mainly cattle and dogs) [47,49,50,51,52]. Similar trends were observed during monitoring of the diets of wolves in Belarus in the 1990s. When a large number of ungulates were observed (elk, roe deer, wild boar), they constituted 88% of the food biomass in the gray wolves’ diet, while domestic animals constituted only 4%. A rapid decline in the ungulate population resulted in significant changes in the diets of wolves. Large ungulates fell to 32% of the biomass in the diet, and domestic animals accounted for 38% of the dietary biomass [52]. Moreover, the wolves ate roe and red deer, whose density in the Vitebsk region was low (3.8 and 0.0 ind./10 km^2^, respectively) after the ASF epidemic outbreak (Table 3). In our opinion, this fact should be considered in conjunction with another observed phenomenon, namely the relation between the share of beaver and elk in the wolf’s diet.

Another phenomenon observed in this study was the relation between the proportion of elk and beaver in the wolves’ diet in the Vitebsk region. The greater the share of elk, the smaller the proportion of beaver. Our results were consistent with previous studies in the Vitebsk region, where wolves increasingly hunted beavers after a decrease in the numbers of elk [52]. Wolves mainly hunt beavers in spring, after the ice cover has subsided, and in autumn. In these two periods, beavers spend more time on land and are therefore more vulnerable to predation [53]. Wolves’ interest in beavers decreases in summer, when predators mainly hunt young ungulates [54]. However, some studies have indicated all-year-round consumption of beaver [18,19,55]; this was explained by mild winters, when a lack of hard ice cover makes beavers more vulnerable to predation. Another factor that makes beavers more vulnerable to wolves is lower water levels (e.g., due to drought or low rainfall). In such conditions, beavers spend more time on land or in shallow water, thus making them more exposed to wolves [19]. In our opinion, however, these weather-related reasons are unlikely to have affected our results, as a similar phenomenon was not noticed in the Grodno region. Both study areas were relatively close to each other, which suggests similar conditions in winter and similar rainfall in the individual years of the study. Thus, why was the beaver’s proportion in the wolves’ diet not related to the proportion of elk in Grodno?

The share of beavers in the wolves’ diet was similar in both regions, with slightly higher values in the Grodno region than in the Vitebsk region (Figure 2). These regions, however, significantly differed in terms of the proportion of elk in the wolves’ diet: in Vitebsk, elk was the most important prey, but in Grodno, it was much less important (Figure 2). In our opinion, these differences could explain the fluctuating proportions of beaver and elk in the wolf’s diet. Wolves prefer to hunt elk calves [16]. Female moose with calves are less mobile, which may make them easier to hunt [56]. Elk, however, show various behavioral adaptations to the presence of wolves, including increased vigilance and aggressive behavior towards predators [15,57,58]. Thus, the effectiveness with which wolves hunt elk may depend on the size of the wolf pack [59]. Beavers can be an important part of the wolf’s diet [5], although consumption of this species may be related to its density. Romański [60] and Moayeri [61] indicated that beaver hunting may also result from the specialization of an individual or family group. Beavers can be attractive prey for lone wolves or small packs, because they are an easier food source compared to ungulates [62,63]. Taking the above into account, we speculate that wolf pack structures have been affected by the results of hunting. Smaller groups or a larger proportion of smaller packs has probably resulted in an increased interest in beavers. We did not possess data on the intensity of wolf hunting by humans; however, we believe that wolf hunting has increased after the wild boar population decline. We speculate that wolves hunted deer more intensively after the ASF epidemic outbreak, and this probably encouraged hunters to exploit wolves more in the Vitebsk region. This speculation could be confirmed by the lack of an increase in livestock depredation in the Vitebsk region after the ASF epidemic outbreak. Such a phenomenon should theoretically occur when a decrease of one of the gray wolf’s main prey is observed [7]. However, less predators effectively means less depredation of farm animals [64]. Increased wolf hunting could also explain the lack of other prey in the wolves’ diet after the ASF epidemic.

## 5. Conclusions

Our study has shown that the drastic decline in the wild boar population after the ASF outbreak triggered a significant change in the diet of the gray wolf. However, this effect was dependent on the importance of wild boar in the wolves’ diet. When this species constituted 10% of their diet, the decrease in its number in the environment did not make any difference for wolves. Wild boar in the diet was replaced by roe and red deer, while the secondary effect of the ASF epidemic outbreak was a fluctuation of elk and beavers in the wolves’ diet. In our opinion, only intensive killing of wolves by humans could explain the resulting dietary fluctuations (between elk and beavers) and the lack of an increase in other prey (including livestock) in the wolves’ diet in the Vitebsk region.

## Figures and Tables

**Table 1:** Wild boar population numbers and hunting intensity (population harvest) in Belarus between 2010 and 2019 (in thousands) in the official data [34].

	2010	2011	2012	2013	2014	2015	2016	2017	2018	2019
Population numbers	69.1	74.0	77.2	80.4	8.6	8.0	5.3	2.8	2.6	2.4
Population harvest	25.9	28.5	29.7	48.1	30.6	17.2	11.7	9.1	7.7	11.4

**Table 2:** Beaver population numbers in Vitebsk and Grodno regions between 2010 and 2019 (in thousands) in the official data [37].

	2010	2011	2012	2013	2014	2015	2016	2017	2018	2019
Vitebsk	13.3	12.7	12.1	12.6	12.4	11.3	10.1	9.8	10.0	10.1
Grodno	8.1	7.6	7.6	8.5	8.6	8.3	7.7	7.6	7.6	8.1

**Table 3:** Ungulate population density (ind./10 km^2^) in selected years in the study areas (Vitebsk and Grodno) [Yanuta, unpublished data].

	Vitebsk				Grodno			
	2011	2013	2015	2017	2012	2016	2017	2018
Wild boar	8.3	11.9	2.7	0.2	27.1	0.9	0.9	0.9
Elk	4.8	4.7	5.0	5.0	3.2	3.6	4.4	4.2
Red deer	0.0	0.0	0.0	0.0	5.4	6.3	6.7	7.1
Roe deer	3.4	4.1	3.4	3.9	28.3	29.6	31.1	29.7

**Table 4:** Number of fecal samples collected in both regions between 2010 and 2020.

	2010	2011	2012	2013	2014	2015	2016	2017	2018	2019	2020	Total
Vitebsk	10	13	15	15	9	10	10	11	12	11	-	116
Grodno	14	9	12	11	10	11	11	12	12	10	9	121

## Data Availability

The data presented in this study are available on request from the corresponding author.

## References

[1] Mech L.D., Karns P.D. (1978). Role of the Wolf in a Deer Decline in the Superior National Forest.

[2] Gasaway W.C., Stephenson R.O., Davis J.L., Shepherd E.K., Burris O.E. (1983). Interrelationships of wolves, prey, and man in interior Alaska. Wildl. Monogr..

[3] Bibikov D., Kudaktin A., Ryabov L. (1985). Synanthropic wolves: Distribution and diet. Zool. Zhurn..

[4] Becker M.S., Garrott R.A., White P.J., Gower C.N., Bergman E.J., Jaffe R. (2008). Chapter 16. Wolf Prey Selection in an Elk-Bison System. Terr. Ecol..

[5] Newsome T.M., Boitani L., Chapron G., Ciucci P., Dickman C.R., Dellinger J.A., López-Bao J.V., Peterson R.O., Shores C.R., Ripple W.J. (2016). Food habits of the world’s grey wolves. Mamm. Rev..

[6] Paquet P.C., Carbyn L.N., Feldhamer G.A., Thompson B.C., Champan J.A. (2003). Gray wolf Canis lupus and allies. Wild Mammals of North America: Biology, Management, and Conservation.

[7] Okarma H. (1995). The trophic ecology of wolves and their predatory role in ungulate communities of forest ecosystems in Europe. Acta Ther..

[8] Begon M., Sait S.M., Thompson D.J. (1996). Predator–Prey cycles with period shifts between two-and three-species systems. Nature.

[9] Jedrzejewska B., Jedrzejewski W., Okarma H., Schmidt K., Zub K., Musiani M. (2000). Prey selection and predation by wolves in Bialowieza Primeval forest, Poland. J. Mammal..

[10] Smith D.W., Drummer T.D., Murphy K.M., Guernsey D.S., Evans S.B. (2004). Winter prey selection and estimation of wolf kill rates in Yellowstone National Park, 1995–2000. J. Wild. Manag..

[11] Mech L.D., Meier T.J., Burch J.W., Adams L.G., Carbyn L.N., Fritts S.H., Seip D.R. (1995). Patterns of prey selection by wolves in Denali National Park, Alaska. Ecology and Conservation of Wolves in a Changing World.

[12] Sand H., Zimmermann B., Wabakken P., Andren H., Pedersen H.C. (2005). Using GPS technology and GIS cluster analyses to estimate kill rates in wolf-ungulate ecosystems. Wildl. Soc. Bull..

[13] Sand H., Wabakken P., Zimmermann B., Johansson Ö., Pedersen H., Liberg O. (2008). Summer kill rates and predation pattern in a wolf moose system: Can we rely on winter estimates?. Oecologia.

[14] Jędrzejewski W., Niedziałkowska M., Hayward M.W., Goszczyński J., Jędrzejewska B., Borowik T., Wojtulewicz M. (2012). Prey choice and diet of wolves related to ungulate communities and wolf subpopulations in Poland. J. Mammal..

[15] Mech L.D., Boitani L. (2003). Wolves: Behavior, Ecology and Conservation.

[16] Hilde K., Hjeljord O. (2003). Wolf predation on moose—A case study using hunter observations. Alces.

[17] Lafferty D.J.R., Belant J.L., White K.S., Womble J.N., Morzillo A.T. (2014). Linking wolf diet to changes in marine and terrestrial prey abundance. Arctic.

[18] Latham A.D.M., Latham M.C., Knopff K.H., Hebblewhite M., Boutin S. (2013). Wolves, white-tailed deer, and beaver: Implications of seasonal prey switching for woodland caribou declines. Ecography.

[19] Sidorovich V.E., Annik S., Schnitzler C., Rotenko I., Holikava Y. (2017). Responses of wolf feeding habits after adverse climatic events in central-western Belarus. Mamm. Biol..

[20] Meriggi A., Lovari S. (1996). A review of wolf predation in southern Europe: Does the wolf prefer wild prey to livestock?. J. Appl. Ecol..

[21] Žunna A., Ozolin Š.J., Pupila A. (2009). Food habits of the wolf *Canis lupus* in Latvia based on stomach analyses. Est. J. Ecol..

[22] Migli D., Youlatos D., Iliopoulos Y. (2005). Winter food habits of wolves in central Greece. J. Biol. Res..

[23] Mori E., Benatti L., Lovari S., Ferretti F. (2017). What does the wild boar mean to the wolf?. Eur. J. Wildl. Res..

[24] Imbert C., Caniglia R., Fabbri E., Milanesi P., Randi E., Serafini M., Torretta E., Meriggi A. (2016). Why do wolves eat livestock?: Factors influencing wolf diet in northern Italy. Biol. Conserv..

[25] Gazzola A., Avanzinelli E., Bertelli I., Tolosano A., Bertotto P., Musso R., Apollonio M. (2007). The role of the wolf in shaping a multi-species ungulate community in Italian western alps. Ital. J. Zool..

[26] Hall A.M. (1971). Ecology of Beaver and Selection of Prey by Wolves in Central Ontario. Master’s Thesis.

[27] Fuller T.K., Keith L.B. (1980). Wolf population dynamics and prey relationships in northeastern Alberta. J. Wildl. Manag..

[28] Gable T.D., Windels S.K., Homkes A.T. (2018). Do wolves hunt freshwater fish in spring as a food source?. Mamm. Biol..

[29] Mattioli L., Apollonio M., Mazzarone V., Centofanti E. (1995). Wolf food habits and wild ungulate availability in the Foreste Casentinesi National Park, Italy. Acta Ther..

[30] Nowak S., Myslajek R.W., Klosinska A. (2011). Diet and prey selection of wolves (*Canis lupus*) recolonising western and central Poland. Mamm. Biol..

[31] Penrith M.-L., Vosloo W. (2009). Review of African swine fever: Transmission, spread and control. J. S. Afr. Vet. Assoc..

[32] Morelle K., Bubnicki J., Churski M., Gryz J., Podgórski T., Kuijper D.P.J. (2020). Disease-Induced Mortality Outweighs Hunting in Causing Wild Boar Population Crash After African Swine Fever Outbreak. Front. Vet. Sci..

[33] Cwynar P., Stojkov J., Wlazlak K. (2019). African swine fever status in Europe. Viruses.

[34] National Statistical Committee of the Republic of Belarus. https://www.belstat.gov.by/upload/iblock/5d4/5d43b8258dd43b307ff59ad3ec654f25.pdf.

[35] Valdmann H., Saarma U. (2020). Winter diet of wolf (*Canis lupus*) after the outbreak of African swine fever and under the severely reduced densities of wild boar (*Sus scrofa*). Hystrix.

[36] Velihurau P.A., Yanuta G.G., Anisimova E.I. (2014). Contemporary biogeografical structure of game ruminants. Proc. Natl. Acad. Sci. Belarus Biol. Ser..

[37] Ministry of Natural Resources and Environmental Protection of the Republic of Belarus https://www.minpriroda.gov.by/ru//.

[38] Priklonsky S.G. (1965). Coefficients to treat the data of winter transect method ofcensus taking of game animals by their traces. Byulleten Moskovskogo Obshchestva Ispytatelei Prirody Ordel Biologii.

[39] Jędrzejewska B., Jędrzejewski W. (1998). Predation in Vertebrate Communities. The Białowieza Primeval Forest as a Case Study.

[40] Pucek Z. (1981). Keys to Vertebrates of Poland.

[41] Debrot S., Mermod C., Fivaz G., Weber J.-M. (1982). Atlas des Poils de Mammiferes d’Europe.

[42] Goszczyński J. (1974). Studies on the food of foxes. Acta Ther..

[43] Teerink B.J. (1991). Atlas and Identification Key Hair of West-European Mammals.

[44] Ansorge H., Kluth G., Hahne S. (2006). Feeding ecology of wolves *Canis lupus* returning to Germany. Acta Ther..

[45] Mattioli L., Capitani C., Avanzinelli E., Bertelli I., Gazzola A., Apollonio M. (2004). Predation by wolf (*Canis lupus*) on roe deer (*Capreolus capreolus*) in north-eastern Apennines. Ital. J. Zool..

[46] Kübarsepp M., Valdmann H. (2003). Winter diet and movements of wolf (*Canislupus*) in Alampedia nature reserve, Estonia. Acta Zool. Litu..

[47] Ciucci P., Artoni L., Crispino F., Tosoni E., Boitani L. (2018). Inter-pack, seasonal and annual variation in prey consumed by wolves in Pollino National Park, southern Italy. Eur. J. Wildl. Res..

[48] Mattioli L., Capitani C., Gazzola A., Scandura M., Apollonio M. (2011). Prey selection and dietary response by wolves in a high-density multi-species ungulate community. Eur. J. Wildl. Res..

[49] Gazzola A., Capitani C., Mattioli L., Apollonio M. (2008). Livestock damage and wolf presence. J. Zool..

[50] Torres R.T., Silva N., Brotas G., Fonseca C. (2015). To eat or not to eat? The diet of the endangered Iberian wolf (*Canis lupus signatus*) in a human-dominated landscape in central Portugal. PLoS ONE.

[51] Capitani C., Chynoweth M., Kusak J., Çoban E., Şekercioglu Ç.H. (2016). Wolf diet in an agricultural landscape of north-eastern Turkey. Mammalia.

[52] Sidorovich V.E., Tikhomirova L.L., Jędrzejewska B. (2003). Wolf Canis lupus numbers, diet and damage to livestock in relation to hunting and ungulate abundance in northeastern Belarus during 1990–2000. Wildl. Biol..

[53] Gable T. (2016). Wolf Predation: Where and How Wolves Kill Beavers, and Confronting the Biases in Scat-Based Diet Studies. Master’s Thesis.

[54] Gable T.D., Windels S.K., Bruggink J.K., Barber-Meyer S.M. (2018). Weekly summer diet of gray wolves (*Canis lupus*) in northeastern Minnesota. Am. Midl. Nat..

[55] Gogan P.J., Route W.T., Olexa E.M., Thomas N., Kuehn D., Podruzny K.M. (2004). Gray Wolves in and Adjacent to Voyageurs National Park, Minnesota: Research and Synthesis, 1987–1991.

[56] Wikenros C., Balogh G., Sand H., Nicholsonm K.L., Mansson J. (2016). Mobility of moose-comparing the effects of wolf predation risk, reproductive status, and seasonality. Ecol. Evol..

[57] Berger J. (1999). Anthropogenic extinction of top carnivores and interspecific animal behaviour: Implications of the rapid decoupling of a web involving wolves, bears, moose and ravens. Proc. R. Soc. Lond. Ser. B Biol. Sci..

[58] White K.S., Berger J. (2001). Antipredator strategies of Alaskan moose: Are maternal trade-offs influenced by offspring activity?. Can. J. Zool..

[59] Gundersen H., Solberg E.J., Wabakken P., Storaas T., Zimmermann B., Andreassen H.P. (2008). Three approaches to estimate wolf *Canis lupus* predation rates on moose Alces alces populations. Eur. J. Wildl. Res..

[60] Romanski M.C. (2010). Estimates of Abundance and Predation—The Population Ecology of Beaver in Isle Royale National Park. Master’s Thesis.

[61] Moayeri M. (2013). Reconstructing the Summer Diet of Wolves in a Complex Multi-ungulate System in Northern Manitoba, Canada. Master’s Thesis.

[62] Benson J.F., Patterson B.R. (2013). Moose (*Alces alces*) predation by eastern coyotes (*Canis latrans*) and eastern coyote × eastern wolf (*Canis latrans × Canis lycaon*) hybrids. Can. J. Zool..

[63] Sand H., Eklund A., Zimmermann B., Wikenros C., Wabakken P. (2016). Prey selection of Scandinavian wolves: Single large or several small?. PLoS ONE.

[64] Wielgus R.B., Peebles K.A. (2014). Effects of Wolf Mortality on Livestock Depredations. PLoS ONE.

